# Energy Availability, Body Composition, and Phase Angle Among Adolescent Artistic Gymnasts During a Competitive Season

**DOI:** 10.3390/nu18030519

**Published:** 2026-02-03

**Authors:** Anneta Grompanopoulou, Antigoni Kypraiou, Dimitrios C. Milosis, Michael Chourdakis, Anatoli Petridou

**Affiliations:** 1 Laboratory of Hygiene, Social & Preventive Medicine and Medical Statistics, Aristotle University of Thessaloniki, 54124 Thessaloniki, Greece; agromp@auth.gr (A.G.); mhourd@auth.gr (M.C.); 2 Laboratory of Evaluation of Human Biological Performance, School of Physical Education and Sport Science at Thessaloniki, Aristotle University of Thessaloniki, 54124 Thessaloniki, Greece; ankypraiou@phed.auth.gr (A.K.); dmylosis@phed.auth.gr (D.C.M.)

**Keywords:** adolescent athletes, artistic gymnasts, body composition, energy intake, energy expenditure, energy availability, phase angle, BIVA

## Abstract

**Background/Objectives**: Energy availability (EA) is associated with Relative Energy Deficiency in Sport syndrome. This study assessed the EA, body composition, and phase angle (φ) of adolescent artistic gymnasts during a competitive season. **Methods**: Thirty non-elite artistic gymnasts aged 11–14 years participated in this cross-sectional study. Anthropometric data were collected and body mass index (BMI) was assessed using the World Health Organization growth charts. Bioelectrical impedance analysis was performed and diet and physical activity were recorded for three days. Dietary and physical activity records were analyzed to estimate energy intake, total energy expenditure (TEE), and exercise energy expenditure, from which energy balance (EB) and EA were calculated. The 95% confidence ellipses of the impedance (Z) vectors were compared with a reference population using the two-sample Hotelling’s T^2^ test. Correlations between variables were examined by Pearson’s or Spearman’s correlation analysis. Statistical significance was set at α = 0.05. **Results**: All participants were classified within the normal BMI category, except for one who was classified as being overweight. Mean (± SD) fat mass, fat-free mass (FFM), and φ were 16.1 ± 3.4%, 83.9 ± 3.4%, and 6.0 ± 0.6°, respectively. The 95% confidence ellipses of Z vectors differed significantly from the reference population. Energy balance was 32 ± 223 kcal/day and EA was 49.2 ± 11.4 kcal/kg FFM/day. Energy availability was significantly correlated with EB, TEE, and body composition variables. **Conclusions**: Adolescent non-elite artistic gymnasts showed no clear indications of LEA and exhibited a normal body composition and φ during the competitive season, consistent with their EA.

## 1. Introduction

Energy availability (EA) is the amount of dietary energy intake (EI), relative to fat-free mass (FFM), that remains available to support physiological functions after accounting for exercise energy expenditure (EEE) [1]. In healthy, physically active individuals, an EA of ≥45 kcal/kg FFM/day is considered adequate [1,2]. Low energy availability (LEA), defined as an EA of ≤30 kcal/kg FFM/day, is considered to be the primary cause of Relative Energy Deficiency in Sport (REDs) syndrome [1]. REDs is a major health concern in athletes, as it can impair several physiological, psychological, and bodily functions [1,3]. 

Apart from EA, the magnitude of total energy expenditure (TEE) may also impact physiological functions. Studies of athletic and non-athletic adults suggest a maximal level of long-term sustained energy expenditure corresponding to a physical activity level (PAL) of approximately 2.5 [4,5,6], beyond which body mass loss commonly occurs. This upper limit has been attributed to constraints in habitual EI capacity [4,5]. A study of adolescent athletes reported a PAL of 1.90—similar to non-athletic adolescents (1.84) [7] and lower than the sustained energy expenditure found in adults.

Bioelectrical impedance analysis (BIA) is a widely used method of assessing body composition. BIA measures the resistance (R) of body tissues and reactance (Xc) to a low-intensity electrical current (≈800 μA) to estimate body composition based on tissue conductivity [8]. Lean tissue, rich in water and electrolytes, conducts electricity well, whereas adipose tissue is a poor conductor. Together, R and Xc are used to calculate impedance (Z), as the vector resultant of R and Xc, and phase angle (φ), as the arctangent of Xc/R [8]. Phase angle serves as an indicator of cellular integrity [9], as intact and well-structured membranes effectively impede and store the electrical charge, resulting in a higher Xc, and consequently, φ. Also, φ values have been positively associated with better cellular health [9], greater muscle mass [10,11,12], and muscle strength [13]. In contrast, lower φ values have been linked to a compromised nutritional status in the pediatric population [14] and malnutrition [15], as well as to various pathological conditions [16,17]. Moreover, by plotting height-standardized R and Xc values, bioelectrical impedance vector analysis (BIVA) provides a non-invasive method for assessing body cell mass and hydration status. In the context of a REDs risk assessment in adolescent athletes, φ and bioelectrical vector position may reflect the nutritional and cellular status resulting from LEA. 

Artistic gymnastics is a popular Olympic sport, in which optimal performance requires speed, a high power-to-body mass ratio, strength, flexibility, coordination, and precise technical execution [18]. To develop these attributes, athletes begin intensive training at a young age, during which major physical and physiological changes take place [19]. Early adolescent competitive artistic gymnasts (≈11 years of age) typically train 20 h per week, with training volume increasing to around 30 h per week in older adolescents (≈17 years of age) [20,21,22,23,24]. Adolescent female artistic gymnasts can expend up to 768 kcal during a 4 h session [24]. The combination of high training loads, increased energy demands for optimal growth, and emphasis on maintaining a low body mass may increase the risk of LEA in this population [25,26,27,28,29].

To our knowledge, few studies have assessed EA in pre-adolescent and adolescent gymnasts and none in artistic gymnasts. Studies on rhythmic and acrobatic gymnasts have reported EA values of below 30 kcal/kg FFM/day [29,30] or between 30 and 45 kcal/kg FFM/day [27,28,29], indicating a potential risk of LEA. These athletes also tend to have a low body mass index (BMI) and fat mass (FM) percentage [27,29,31]. In contrast, studies in adolescents artistic gymnasts have reported a wide range of body composition values [25,26], likely reflecting differences in training status, as well as high φ values (around 7°) [25].

The presentation above shows that no studies have evaluated EA in adolescent artistic gymnasts, while research examining body composition and φ in this population remains limited. Therefore, the aim of the present study was to estimate EI, TEE, and EEE, and consequently assess EB and EA in non-elite adolescent artistic gymnasts during a competitive season. Additionally, this study aimed to assess body composition and φ, as complementary indicators of nutritional status, and to examine their associations with EA. We hypothesized that EA would vary among adolescent artistic gymnasts and that a proportion of athletes might present values below the proposed adequacy thresholds. Furthermore, we hypothesized that EA would be associated with body composition variables and φ.

## 2. Materials and Methods

### 2.1. Participants and Ethics

This study included 30 adolescents artistic gymnasts (24 girls and 6 boys) from various sport clubs in Thessaloniki, Greece. The sample size was determined through an a priori power analysis conducted using G*Power software (version 3.1.9.2, Kiel University, Kiel, Germany). The analysis indicated that, assuming a statistical power of 0.8, a significance level of α = 0.05, low data variability, and a moderate-to-high correlation coefficient (r > 0.4), a total of 30 athletes were required. Inclusion criteria were as follows: (1) age between 11 and 14 years, (2) at least two years of continuous training in artistic gymnastics, (3) participation in national-level competitions, and (4) preparation for competition during the study period. Exclusion criteria were as follows: (1) any chronic disease affecting EI or expenditure or (2) musculoskeletal injuries that could limit training during competitive preparation.

All participants were classified as Tier 2 (trained/developmental), based on training age, weekly training volume, and participation in national-level competitions, following the framework proposed by McKay et al. [32]. Competitive level was further characterized by using individual all-around scores derived from the FIG Code of Points [33] regulations specific to each age category. Scores were obtained from an international club competition held after the competitive preparation period in which the artistic gymnasts of this study participated. Mean scores of the study participants were 37.00 for girls and 60.24 for boys, whereas the maximum scores achieved by the participants in this competition were 46.25 for girls and 68.77 for boys.

Participants and their parents received oral and written information about the purpose of the study, procedures, potential benefits of participation, and data-protection measures, after which written informed consent was obtained. The research was approved by the Ethics Committee of the School of Medicine, Aristotle University of Thessaloniki (approval number 82/2024). 

### 2.2. Study Design 

This was an observational, cross-sectional study conducted during a competitive season. Demographic and anthropometric data were collected, and body composition was assessed using the BIA. Participants also recorded their dietary intake, including supplements, and physical activity for three days.

### 2.3. Demographic Characteristics

Demographic data were collected through interviews, during which a structured questionnaire was completed. Recorded information included date of birth, training age, weekly training hours, and for girls, age at menarche. Apart from age at menarche in girls, biological maturation status (e.g., pubertal stage or hormonal markers) was not directly assessed.

### 2.4. Anthropometrics and Body Composition

Body mass was measured to the nearest 0.1 kg with minimal clothing using an electronic scale (Seca, Hamburg, Germany). Body height was measured to the nearest 0.01 m without shoes using a stadiometer. BMI was then calculated and evaluated using the BMI-for-age growth charts for children and adolescents aged 5–19 years, provided by the World Health Organization (WHO).

Body composition was assessed through a multi-frequency BIA using a Bodystat QuadScan 4000 device (Douglas, UK). Measurements were carried out with participants lying supine, with arms and legs slightly apart. Electrodes were placed on the right hand (at the wrist and above the middle finger) and right foot (at the ankle and above the second toe), as per the manufacturer’s instructions. An electrical current at 5, 50, 100, and 200 kHz was applied. The following variables were obtained: FM (kg and %), FFM (kg and %), total body water (TBW, L and %), extracellular water (ECW, L and %), intracellular water (L and %), third-space water (L), body cell mass (BCM, kg), R (Ω), Xc (Ω), φ (°), and Z (Ω). Resistance and Xc were standardized for the participants’ height and BIVA was performed. All measurements were conducted prior to training sessions, after at least 12 h without vigorous exercise and at least three hours in the fasted state. 

### 2.5. Analysis of Energy Intake

Energy intake was estimated through three-day food diaries (two weekdays and one weekend day) completed by the participants with parental assistance. Parents received online training on accurate dietary recording and written instructions with portion-size guidance. Additionally, an example of a completed daily food record was included in the food diaries to facilitate proper reporting. 

The dietary records were analyzed using the online Cronometer application (Revelstoke, BC, Canada, https://cronometer.com/index.html, accessed on 31 January 2026) with nutritional data sourced from the FoodData Central database provided by the U.S. Department of Agriculture. Energy contribution from supplements was verified and calculated based on product label information.

### 2.6. Analysis of Energy Expenditure

Resting energy expenditure (REE) was estimated using the age- and sex-specific Schofield equation [34].

Physical-activity energy expenditure was determined from three-day physical activity diaries completed concurrently with the dietary records. Participants, with parental assistance, recorded all daily activities and the duration of each over 24 h. Detailed instructions for accurate recording were provided during an online session with parents and in-person meetings with coaches. Furthermore, an example of a completed daily physical activity record was included in the diaries. Activity data were analyzed using the youth metabolic equivalent of task (MET) values from the Youth Compendium of Physical Activities [35]. Although this database includes a smaller variety of activities than the Compendium of Physical Activities for adults [36], it provides age-specific data, ensuring greater accuracy in estimation. When a recorded activity was not listed in the youth database, a comparable activity was selected.

Total energy expenditure was calculated by determining a weighted average MET, based on the duration and MET value of each activity, multiplying this value by REE, and adding 10% to account for energy expenditure from diet-induced thermogenesis.

To verify EEE, two researchers independently observed and recorded one training session of four randomly selected athletes from different sport clubs. The observations were conducted under blinded conditions; neither the athletes nor the coaches were aware that the training session was being observed and recorded. Each researcher independently observed one athlete at a time, recording the exact duration of each exercise, estimated intensity, and duration of rest intervals. EEE values derived from direct observation were lower and were therefore taken into account, as in practice there are inevitably periods of inactivity during training sessions. 

### 2.7. Energy Balance and Energy Availability

The energy balance (EB) on each of the three days of dietary and physical activity recording was calculated as the difference between EI and TEE. Energy availability was calculated according to the formula provided by the International Olympic Committee [1], using the average EI and EEE of the two weekdays that included gymnastics training: EA = (EI − EEE)/FFM. 

### 2.8. Assessment of Dietary Underreporting 

To assess potential underreporting of dietary intake, the difference between each participant’s estimated EI and the reference EI values provided by the European Food Safety Authority (EFSA)—based on age, sex, and Physical Activity Level (PAL) [37]—was calculated. The PAL for each participant was determined as the mean ratio of TEE to REE across the three days of dietary and physical activity recording. When underreporting (defined as a negative difference between estimated and reference EI) was identified, the participant’s EI was adjusted to the reference EI. Subsequently, EB (calculated as the corrected EI minus TEE) and EA (using the corrected EI in the corresponding formula) were recalculated.

### 2.9. Statistical Analysis

Data are presented as the mean ± standard deviation (SD). Correlations between conceptually relevant study variables were examined by Pearson’s or Spearman’s correlation analysis, depending on whether data distribution did not differ or differed from normal based on the Shapiro–Wilk test. For BIVA, Xc and R, measured at 50 kHz, were standardized to the participants’ height. Subsequently, the 95% confidence ellipses of the participants in the present study were compared with those of a reference population [38] using the two-sample Hotelling’s T^2^ test. Vector analysis was performed using the BIVA 2002 software [39]. The level of statistical significance was set at α = 0.05. All statistical analyses were conducted using SPSS version 29.0 (IBM, Armonk, NY, USA).

## 3. Results

**Figure 1:**
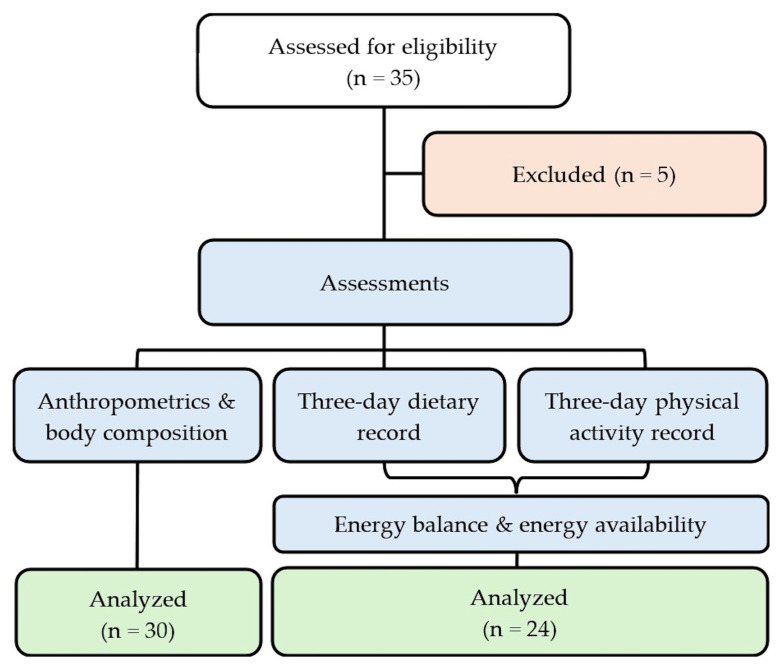
Participant flow diagram.

A total of 35 gymnasts were approached and assessed for eligibility to participate in the study. Five were excluded because they did not meet the inclusion criteria related to gymnastics type and age or met the exclusion criteria such as injury or illness. Anthropometric and body composition measurements were conducted on the remaining 30 athletes (24 girls and 6 boys). As descriptive comparisons of the study variables between girls and boys did not reveal meaningful differences, both were included in the analyses. Of those 30 athletes, 24 (20 girls and 4 boys) completed the three-day dietary and physical activity records, while the remaining 6 did not return their records and were therefore included in the anthropometric and body composition analysis only (Figure 1).

### 3.1. Demographic Characteristics

The demographic characteristics of the participants are shown in Table 1. Of the 24 girls, 10 reported having reached menarche at the age of 12.6 ± 0.7 years. The age of the 10 athletes who had menstruated was 13.3 ± 0.7 years, while the mean age of those who had not yet menstruated was 11.9 ± 0.9 years.

### 3.2. Anthropometrics and Body Composition

Table 2 presents the anthropometric and body composition variables of the participants. 

**Figure 2:**
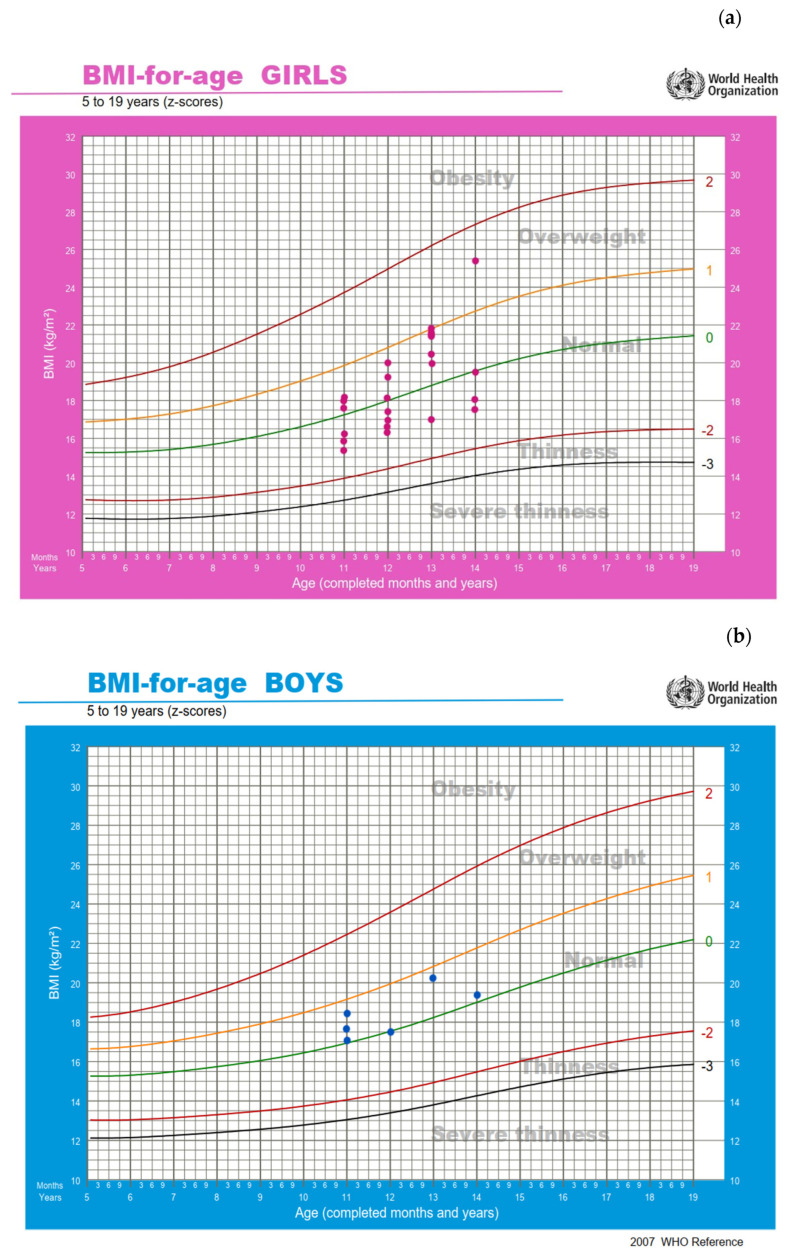
Position of participants on the WHO BMI-for-age growth charts: (**a**) girls (*n* = 24) and (**b**) boys (*n* = 6) [40]. Pink dots represent female adolescent artistic gymnasts, and blue dots represent male adolescent artistic gymnasts.

All participants were classified as being in the normal BMI category according to the WHO growth charts, except for one girl who was classified as being overweight. The BMI z-score value was 0.10 ± 0.70 for all participants (*n* = 30) and 0.11 ± 0.75 for the participants with complete energy data (*n* = 24, Figure 2).

**Figure 3:**
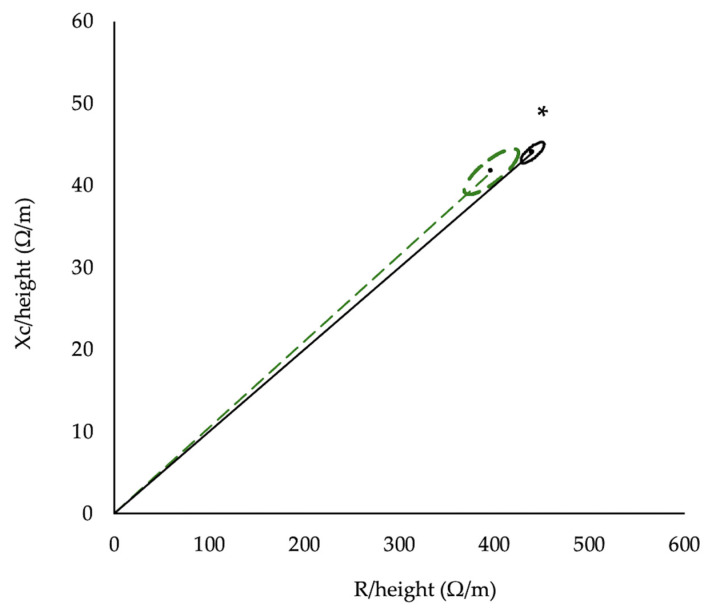
95% confidence ellipses for the mean bioelectrical impedance vectors of participants in the present study (dashed line) and the reference population (solid line) [38]. Xc: reactance, R: resistance. Analysis was performed using Hotelling’s T^2^ test. * Significant difference, *p* = 0.0004.

A statistically significant difference was found between the Z vectors of the participants in the present study and those of a reference population [38] (T^2^ = 16.3, F = 8.1, *p* = 0.0004, Mahalanobis D = 0.82, Figure 3).

### 3.3. Energy Balance and Energy Availability 

**Figure 4:**
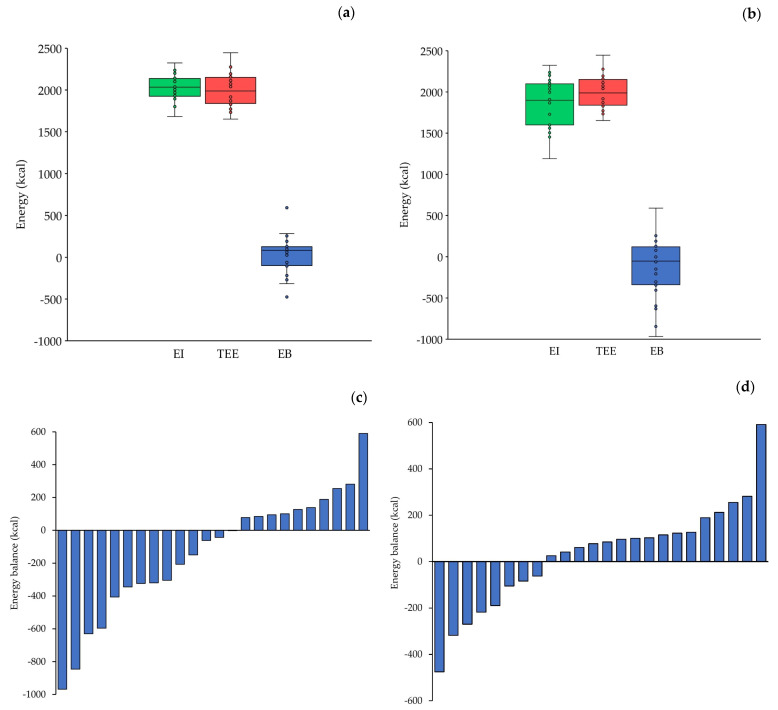
(**a**,**b**) Boxplots of daily energy intake (EI), total energy expenditure (TEE), and energy balance (EB) of the participants (*n* = 24), averaged over 3 days, (**a**) before adjustment for dietary underreporting and (**b**) after adjustment for dietary underreporting. Each box represents the interquartile range, and the center line represents the median. Whiskers are extended to the most extreme data point that is no more than 1.5 times the interquartile range from the edge of the box (Tukey style). Dots represent individual values. (**c**,**d**) Individual EB of the participants, (**c**) before adjustment for dietary underreporting and (**d**) after adjustment for dietary underreporting.

Underreporting of dietary intake was identified in 63% of the participants, with an average underreporting level of 17%. The participants’ total daily EI was 1865 ± 295 kcal (Figure 4a) and increased to 2033 ± 148 kcal after adjustment for underreporting (Figure 4b). The daily REE was 1290 ± 134 kcal, EEE was 396 ± 87 kcal, and TEE reached 2001 ± 204 kcal (Figure 4a,b). The PAL was 1.56 ± 0.06. 

The daily EB was initially negative (–135 ± 374 kcal, Figure 4a) but became positive after adjustment for underreporting (32 ± 223 kcal, Figure 4b). Specifically, 14 out of the 24 athletes (58%) initially exhibited a negative EB (Figure 4c), whereas, after adjustment for underreporting, 8 of the 24 athletes (33%) retained a negative EB (Figure 4d).

**Figure 5:**
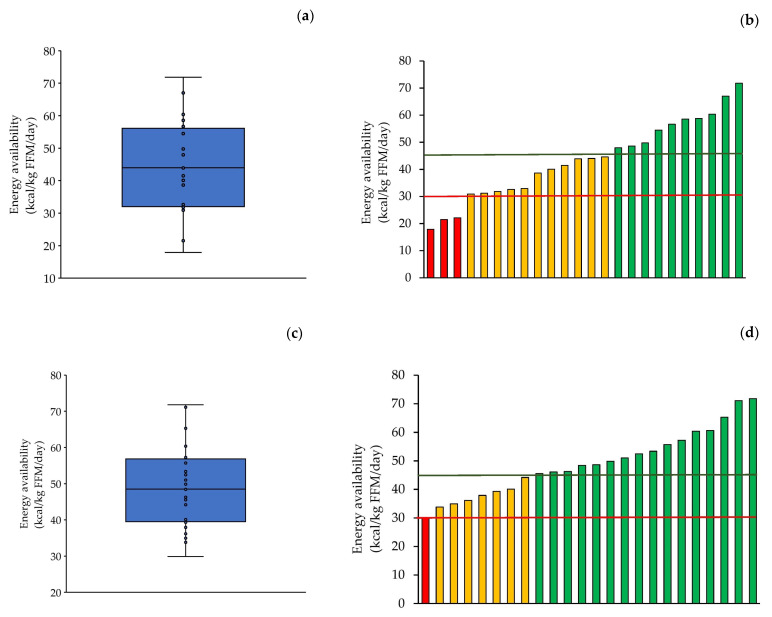
(**a**,**c**) Energy availability (EA) of the participants (*n* = 24), averaged over 2 days, (**a**) before adjustment for dietary underreporting and (**c**) after adjustment for dietary underreporting. Each box represents the interquartile range, and the center line represents the median. Whiskers are extended to the most extreme data point that is no more than 1.5 times the interquartile range from the edge of the box (Tukey style). Dots represent individual values. (**b**,**d**) Individual EA of the participants, (**b**) before adjustment for dietary underreporting and (**d**) after adjustment for dietary underreporting. Red bars represent participants with EA below the defined threshold for low energy availability (red line), orange bars represent participants with EA between the low and adequate EA thresholds (green line), and green bars represent participants with adequate EA.

The participants’ EA was 43.7 ± 14.4 kcal/kg FFM/day (Figure 5a) and increased to 49.2 ± 11.4 kcal/kg FFM/day after adjusting EI for underreporting (Figure 5c). Before adjustment for underreporting, two girls and one boy had EA values below 30 kcal/kg FFM/day, and eleven girls had values below 45 kcal/kg FFM/day (Figure 5b). Following adjustment for underreporting, one girl remained below 30 kcal/kg FFM/day, whereas six girls and one boy had EA values below 45 kcal/kg FFM/day (Figure 5d). 

### 3.4. Correlations

Correlation analysis revealed significant correlations between EA and BCM, R, Xc, EB (averaged over the two weekdays with gymnastics training), and TEE (averaged over the two weekdays with gymnastics training), both before and after adjustment for the underreporting of dietary intake (Table 3 and Table 4, respectively). Additionally, after adjustment for underreporting, EA was significantly correlated with FM in kg (Table 4). Phase angle was significantly correlated with body weight, BMI, FM (kg), FFM (kg), TBW, and ECW (Table 5).

## 4. Discussion

In the present study, we found no clear indications of LEA in non-elite adolescent artistic gymnasts during a competitive season. Additionally, anthropometric and body composition variables, as well as φ, were within the normal range. Within the range of normal EA observed in the present study, φ did not appear to be associated with EA.

One of the consequences of LEA, and a severe indicator of REDs syndrome, is primary amenorrhea [1]. In the present study, around half of the girls had reached menarche at a mean age of 12.6 years, which is slightly earlier than that reported in adolescent rhythmic gymnasts [41,42]. Additionally, BMI values were within the normal range, further supporting appropriate physical development. These findings are consistent with the BMI values reported in similar populations [27,29,30,41,43]. 

Regarding body composition, no specific and widely accepted normative values exist for children and adolescents. The FM percentage observed in the present study (16.1%) was similar to values reported in healthy Polish and Greek adolescents [44,45] and Bulgarian female artistic gymnasts [43]. Lower FM percentages have been reported in elite artistic [46], acrobatic [27], and rhythmic gymnasts [29,31], likely reflecting higher training volumes and lower EI. In contrast, higher values (up to 22.9%) have been described in artistic and acrobatic gymnasts [23,30], possibly attributable to the different training level and/or body composition method used (dual-energy X-ray absorptiometry). Concerning FFM, only two studies have reported the FFM percentage in gymnastic athletes [29,30]: Villa et al. found values comparable to those in the present study [29], whereas Besor et al. reported values that were approximately 5% lower [30].

Regarding φ, it was slightly higher (by 0.2–0.3°) than values reported in healthy Caucasian children of a similar age [38] and lower than those reported in adolescent rhythmic gymnasts [41], possibly due to the older age of the participants (≈15 years). BIVA further demonstrated a leftward shift of the impedance vector relative to reference data of healthy 12-year-old children [38], consistent with studies in youth athletes from various sports [47,48,49], suggesting an increased BCM [50]. Indeed, we found a BCM of about 20 kg, which is about 4 kg higher than the values reported in healthy adolescents of a similar age [51].

The percentage of dietary underreporting identified in this study aligns with findings from studies involving children, adolescents, and adults [52,53,54]. Underreporting may arise from unconscious reasons, such as difficulty in estimating portion sizes, inaccurate measurements, or forgetting to record consumed foods, and conscious reasons, including feelings of guilt, the desire to present a more disciplined image, or a lack of motivation.

Our findings indicate that the adolescent artistic gymnasts had no clear indications of LEA, as reflected by the adequate EA and the nearly neutral EB. This contrasts with reports of low EB, LEA, and, in some cases, menstrual irregularities in adolescent athletes participating in rhythmic and acrobatic gymnastics [27,29,30,41]. This discrepancy may be partly explained by the younger age and the lower training load of the participants in the present study, differences in the type of gymnastics, as well as methodological issues related to dietary intake assessment. Importantly, when EI was not adjusted for underreporting, EB was negative and most participants exhibited EA values slightly below the proposed adequate reference value for sedentary and recreationally active adults [1,2]. This finding highlights the sensitivity of EB and EA estimates to dietary reporting accuracy and indicates that these results should be interpreted with caution. Training characteristics further support the observed differences between studies: Gοulart et al. [24] reported a training volume of approximately 20 h per week in artistic gymnasts aged around 11 years, which is consistent with the training volume of the athletes in the present study, whereas studies of older adolescent artistic gymnasts (15–17 years) have reported higher training volumes, at around 30 h per week [20,21,22,23]. In line with this, the relatively low PAL (1.6) observed in our athletes is well below the PAL of 2.5, which is generally considered the upper limit at which it becomes difficult to fully compensate for energy expenditure through EI [4,5,6].

Τhe negative correlation between EA and FM (kg) could partly reflect the impact of LEA on energy metabolism and regulation. Specifically, sustained LEA can reduce the resting metabolic rate, induce hypothyroidism, and increase cortisol [55], all of which may negatively affect physiological function. Indeed, studies have reported a higher FM in female adolescent athletes with EA of <30 kcal/kg FFM/day than those whose EA exceeded this threshold [56,57]. One possible explanation is that individuals with higher body fat have been reported to exhibit greater dietary underreporting compared with individuals with lower body fat [56].

The negative correlation between EA and BCM may partly reflect the mathematical expression of EA relative to FFM, given that BCM represents the protein-rich, metabolically active compartment of FFM [8]. Thus, for similar EI and EEE, athletes with higher BCM may present a lower EA due to a larger denominator. A physiological interpretation consistent with the observed negative correlation is also plausible, as athletes with a higher BCM may have greater overall energy requirements and/or EEE; if EI does not increase proportionally, EA may be lower. Similarly, the positive correlation between EA and R may partly reflect the inverse relationship between R and FFM, as R decreases with increasing FFM. The positive correlation between EA and Xc may indicate the influence of EA on cellular integrity, as adequate EI is required to support cell membrane structure and function. Finally, the negative correlation between EA and TEE and its positive correlation with EB are largely influenced by shared calculation components and should therefore be interpreted with caution.

Regarding φ, its significant correlations with other anthropometric and body composition variables are consistent with the findings of previous research. Specifically, body weight, BMI, FM, FFM, TBW, and ECW have all been identified as determinants of φ [58,59]. In contrast, the absence of an association between φ and EA—both before and after adjustment for dietary underreporting—suggests that φ may not be directly linked to EA, at least within the range of normal EA observed in the present study. Given the absence of correction for multiple correlation tests and the cross-sectional design of the study, all of the aforementioned associations do not allow causal interpretation, and the findings should be interpreted as exploratory and hypothesis generating.

The main limitations of the present study are as follows: First, the sample included a small number of boys, as participation in most sport clubs in Thessaloniki, Greece is predominately girls. Consequently, the findings primarily represent girls, limiting the interpretation of results by sex. Second, biological maturation stage and hormonal status were not assessed, which limits the ability to disentangle the effects of dietary intake from those related to growth and pubertal development. However, EA was expressed relative to FFM, which partially accounts for individual differences in growth-related lean tissue accretion and metabolic demand. Third, 20% of the athletes did not complete the three-day dietary intake and physical activity records, reducing the available data for estimating EB and EA. In addition, dietary intake assessment is subject to reporting inaccuracies. To address this issue, adjustments were applied in cases of dietary underreporting, assuming the underestimation of portion sizes rather than omission of foods. Moreover, the adjustment of EI based on EFSA reference values relies on assumptions about underreporting that may not apply to all participants. To allow transparent interpretation, we present both unadjusted and adjusted EB and EA values. Fourth, EEE was estimated based on the observations of a representative subset of athletes and training sessions, rather than all participants and sessions. Additionally, energy expenditure during gymnastics apparatus exercises was estimated using standardized MET values, which may not fully reflect the intensity of all movements; however, these exercises represented a small proportion of the total training time and were therefore unlikely to substantially affect EA estimates.

The importance of the present study lies in the first-time estimation of EA in adolescent artistic gymnasts, particularly non-elite athletes—a population that represents a large segment of the sporting community but remains underrepresented in the predominantly elite-focused literature. From a practical perspective, the observed interindividual variability in EB and EA supports a personalized approach to nutritional and training strategies. Such an approach may assist coaches, practitioners, and parents in monitoring energy intake relative to training demands and in identifying athletes who may require closer nutritional supervision during the competitive season. Further studies are recommended in young and older athletes who typically engage in more demanding training programs, since there are no proposed EA reference values for non-adults, competitive athletes, or sport-specific contexts. Moreover, future research should address not only EA, but also additional factors proposed to contribute to the risk of developing REDs syndrome [60]. 

## 5. Conclusions

In conclusion, adolescent non-elite artistic gymnasts showed no clear indications of LEA and exhibited normal BMI and body composition values during a competitive season. After adjustment for estimated dietary underreporting, EB and EA were within the ranges considered sufficient to support physiological functions in all but one athlete. Also, φ was not directly associated with EA, at least within the range of normal EA observed in the present study.

## Figures and Tables

**Table 1:** Demographic characteristics (mean ± SD) of all participants (*n* = 30) and of those with complete energy intake and energy expenditure data (*n* = 24).

Variable	*n* = 30	*n* = 24
Age (years)	12.4 ± 1.1	12.4 ± 1.1
Training age (years)	7.6 ± 1.7	7.7 ± 1.7
Training duration (h/week)	17.1 ± 3.9	17.4 ± 3.9

**Table 2:** Anthropometrics, body composition, and φ of all participants (*n* = 30) and of those with complete energy intake and energy expenditure data (*n* = 24).

Variable	*n* = 30	*n* = 24
Weight (kg)	42.5 ± 8.9	42.6 ± 9.3
Height (m)	1.50 ± 0.09	1.50 ± 0.09
Body mass index (kg/m^2^)	18.7 ± 2.2	18.8 ± 2.4
Fat mass (kg)	7.0 ± 2.5	6.9 ± 2.6
Fat mass (%)	16.1 ± 3.4	16.0 ± 3.7
Fat-free mass (kg)	35.5 ± 7.1	35.7 ± 7.4
Fat-free mass (%)	83.9 ± 3.4	84.0 ± 3.7
Total body water (L)	27.0 ± 5.2	27.1 ± 5.5
Total body water (%)	63.7 ± 2.7	63.8 ± 2.9
Extracellular water (L)	12.7 ± 1.8	12.7 ± 1.9
Extracellular water (%)	30.3 ± 2.4	30.3 ± 2.5
Intracellular water (L)	13.9 ± 2.8	13.9 ± 2.9
Intracellular water (%)	33.1 ± 3.6	32.8 ± 3.3
Third-space water (L)	0.3 ± 1.7	0.5 ± 1.6
Body cell mass (kg)	19.9 ± 3.9	19.9 ± 4.1
Resistance (Ω)	589 ± 59	586 ± 63
Reactance (Ω)	62.0 ± 5.6	61.9 ± 6.2
Phase angle (°)	6.0 ± 0.6	6.1 ± 0.6
Impedance at 5 kHz (Ω)	669 ± 62	666 ± 66
Impedance at 50 kHz (Ω)	593 ± 59	589 ± 63
Impedance at 100 kHz (Ω)	562 ± 57	559 ± 61
Impedance at 200 kHz (Ω)	535 ± 56	531 ± 59

**Table 3:** Correlation analysis between energy availability and other conceptually relevant study variables before adjustment for underreporting of dietary intake in adolescent artistic gymnasts during the competitive season (*n* = 24).

	*r* or ρ	*p*
Fat mass (kg)	−0.365 ^a^	0.080
Fat mass (%)	0.002	0.993
Body cell mass (kg)	−0.576	**0.003**
Resistance (Ω)	0.643	**<0.001**
Reactance (Ω)	0.476	**0.019**
Phase angle (°)	−0.231	0.276
Total energy expenditure (kcal)	−0.490	**0.015**
Energy balance (kcal)	0.912	**<0.001**

Boldface indicates significant correlations (*p* < 0.05). ^a^ Spearman’s ρ. All other values are Pearson’s *r*.

**Table 4:** Correlation analysis between energy availability and other conceptually relevant study variables after adjustment for underreporting of dietary intake in adolescent artistic gymnasts during the competitive season (*n* = 24).

	*r* or ρ	*p*
Fat mass (kg)	−0.418 ^a^	**0.042**
Fat mass (%)	−0.023	0.913
Body cell mass (kg)	−0.659	**<0.001**
Resistance (Ω)	0.770	**<0.001**
Reactance (Ω)	0.497	**0.014**
Phase angle (°)	−0.345	0.099
Total energy expenditure (kcal)	−0.571	**0.004**
Energy balance (kcal)	0.836	**<0.001**

Boldface indicates significant correlations (*p* < 0.05). ^a^ Spearman’s ρ. All other values are Pearson’s *r*.

**Table 5:** Correlation analysis of phase angle with anthropometric and body composition variables in adolescent artistic gymnasts during the competitive season (*n* = 30).

	*r* or ρ	*p*
Weight (kg)	0.463	**0.010**
Body mass index (kg/m^2^)	0.588 ^a^	**<0.001**
Fat mass (kg)	0.428 ^a^	**0.018**
Fat mass (%)	0.351	0.057
Fat-free mass (kg)	0.400	**0.029**
Fat-free mass (%)	−0.351	0.057
Total body water (L)	0.395	**0.031**
Total body water (%)	−0.409	**0.025**
Extracellular water (L)	0.364	**0.048**
Extracellular water (%)	−0.497	**0.005**
Intracellular water (L)	0.341	0.065
Intracellular water (%)	−0.230 ^a^	0.221
Body cell mass (kg)	0.340	0.066

Boldface indicates significant correlations (*p* < 0.05). ^a^ Spearman’s ρ. All other values are Pearson’s r.

## Data Availability

The original data presented in the study are openly available in the repository of Hellenic Academic Research Data Management Initiative at https://doi.org/10.26255/heal.3hns-8f0a.

## Abbreviations

The following abbreviations are used in this manuscript:

BCM	Body cell mass
BIA	Bioelectrical impedance analysis
BIVA	Bioelectrical impedance vector analysis
BMI	Body mass index
EA	Energy availability
EB	Energy balance
ECW	Extracellular water
EEE	Exercise energy expenditure
EFSA	European Food Safety Authority
EI	Energy intake
FFM	Fat-free mass
FM	Fat mass
LEA	Low energy availability
MET	Metabolic equivalent of task
PAL	Physical activity level
R	Resistance
REDs	Relative Energy Deficiency in Sport
REE	Resting energy expenditure
SD	Standard deviation
TBW	Total body water
TEE	Total energy expenditure
WHO	World Health Organization
Xc	Reactance
Ζ	Impedance
φ	Phase angle
